# Monolithic Structure-Optical Fiber Sensor with Temperature Compensation for Pressure Measurement

**DOI:** 10.3390/ma12040552

**Published:** 2019-02-13

**Authors:** Wenhua Wang, Xinlei Zhou, Weina Wu, Jihua Chen, Shenlong He, Weifeng Guo, Junbin Gao, Shaoxin Huang, Xuanhua Chen

**Affiliations:** 1 School of Electronic and Information Engineering, Guangdong Ocean University, Zhanjiang 524088, China; xcyjth0751@sina.com (J.C.); hsl576403215@163.com (S.H.); m18320310291@163.com (W.G.); cngjbino@sina.com (J.G.); 13126053319@163.com (S.H.); 13528181760@163.com (X.C.); 2 School of Optoelectronic Engineering and Instrumentation Science, Dalian University of Technology, Dalian 116024, China; xlzhou@dlut.edu.cn; 3 School of mathematics and computer science, Guangdong Ocean University, Zhanjiang 524088, China; suona140@163.com

**Keywords:** optical fiber sensor, laser welding, pressure, Fabry-Perot interferometer, fiber Bragg grating

## Abstract

In this paper, an optical fiber pressure sensor cascading a diaphragm-assisted Fabry-Perot interferometer (FPI) and a fiber Bragg grating (FBG) is proposed and demonstrated. The sensor comprises an optical fiber, a fused-silica ferrule, and a fused-silica diaphragm. We use a femtosecond laser firstly to fabricate a pit on the end face of the ferrule and then investigate the laser heat conduction welding and deep penetration welding technology for manufacturing the seepage pressure sensor of the all-fused-silica material. We develop a sensor based on a monolithic structured FPI without adhesive bonding by means of all-laser-welding. The pressure characteristics of the sensor have good linearity at different temperatures. Also, the monolithic structured sensor possesses excellent resolution, hysteresis, and long-term stability. The environmental temperature obtained by the FBG is employed to compensate for the difference in seepage pressure at different temperatures, and the difference in seepage pressure responses at different temperatures is shown to be very small after temperature compensation.

## 1. Introduction

The failure of a levee can cause flood damage, posing a serious threat to human residential areas and infrastructures. Seepage is one of the main factors leading to the ultimate failure of a levee. Also, large hydropower projects mostly adopt a scheme for dams of tens of meters or even more than 100 m in height. Changes in the seepage field will inevitably cause a change in seepage pressure, which will change the external load acting on the body of levees and dams, thus changing the distribution of the stress field of levees and dams. This leads to hydraulic fractures, deformation, and the instability of levees and dams in their entirety. Previous research has proposed the seepage-stress coupling model to analyze the effect of seepage on the safety of levees and dams [1,2]. The well-known break event of the Teton dam was the result of seepage failure [3]. Therefore, seepage pressure monitoring is a very important element for the safety and the stability of levees and dams.

Seepage pressure monitoring has changed to the precise measurement of automation and the construction of the levees and the dams with smart materials. The traditional monitoring method involves the use of the differential resistance electronic sensor, which is sensitive to electromagnetic disturbance, is moisture-proof, and is resistant to conductivity and corrosion problems. However, the requirements for waterproofing and other electrical parameters of wires and devices are very strict, and their long-term stability is poor. Therefore, it is very challenging to achieve high resolution and high accuracy measurement for the seepage pressure of levees and dams. The technology used for the seepage pressure monitoring of levees and dams is beginning to move towards optical fiber sensing technology so as to achieve the purpose of health monitoring of levees and dams. Optical fiber sensors are excellent candidates for constructing smart levees and dams because of many advantages of optical fiber sensing technology, such as their intrinsic safety, good insulation performance, strong immunity to electromagnetic interference, high sensitivity, high temperature resistance, and excellent distributed monitoring ability, and they are an effective way to address the seepage pressure monitoring problem of levees and dams. The Fabry–Perot interferometer (FPI) optical fiber pressure sensors have drawn great attention due to their high sensitivity and anti-polarization fading characteristic, and they have been extensively investigated in the fields of pressure [4,5,6] and strain [6,7,8,9] monitoring since 1988 [10]. The diaphragm-assisted FPI optical fiber sensors, which were first reported in 1991 [11], are the one of the important types of FPI sensor, and they have become the research focus in the field of acoustic wave [12,13], photo-acoustic spectroscopy [14,15], and for the improvement of performance parameters for dynamic and static pressure measurements [16,17,18,19,20]. The diaphragm-assisted configurations are more suitable for pressure measurement fields requiring high sensitivity and high resolution. However, diaphragm-assisted FPI optical fiber sensors are usually composed of several materials or several elements [15,17,18,20]. A mismatch in the coefficient of thermal expansion (CTE) between materials will cause undesirable stresses to press against each other. The stress will degrade the performance of the FPI sensors and even cause their failure. Also, even if the diaphragm-assisted FPI optical fiber sensor configurations are made up of a kind of material, the current common bonding adhesive of epoxy [21] will influence the performance of sensor, and the adhesive of epoxy will decompose at high temperatures. Additionally, this will cause a large temperature dependence due to the adhesive of epoxy having a different CTE from the materials of the sensor. In order to improve performance, some of the FPI sensors are manufactured in a vacuum environment [22,23] or are composed of monolithic material [24,25]. The requirement of the fabrication in a vacuum is relatively high. The monolithic sapphire in literature [24] can eliminate the mismatch in CTE, but it is more expensive than fused-silica. Laser welding provides a technical method for the fabrication of optical fiber sensors based on monolithic fused-silica. The temperature dependence of optical fiber pressure sensors is quite significant and will lead to a relatively large measurement error. The specific temperature compensation method by fiber Bragg grating (FBG) was described in a previous publication [26] by our group. It is well known that FBG has a good temperature response, while the FPI sensor performs well in terms of sensitivity to pressure. Hence, the multiplexing of FBG and FPI can effectively reduce the error in pressure measurement. Additionally, to achieve higher resolution, the demodulation algorithm for demodulating FP cavity length has become a research focus, and a Vernier demodulation algorithm was reported by our group [27].

In this paper, an optical fiber pressure sensor cascading a diaphragm-assisted FPI and an FBG is proposed and demonstrated. Fabrication of all-laser-welding of FPI leads to the formation of a monolithic structure of all-fused-silica materials as the seepage pressure sensor. The FPI possesses an open FP cavity due to the CO_2_ laser deep penetration point welding between the single mode optical fiber (SMF) and the fused-silica ferrule, which eliminates the undesirable pressure on the inside surface of the diaphragm and improves the temperature stability of the sensors. The fused-silica diaphragm is welded on the ferrule end face by CO_2_ laser heat conduction welding. The FPI manufactured by all-laser-welding has a good performance and stability, because no adhesive of epoxy is involved. In order to avoid the measurement error induced by temperature change, FBG is spliced to be very close to the FP cavity. The temperature compensation by FBG is carried out. 

## 2. Operating Principles and Fabrication of Sensor

### 2.1. Operating Principles

**Figure 1:**
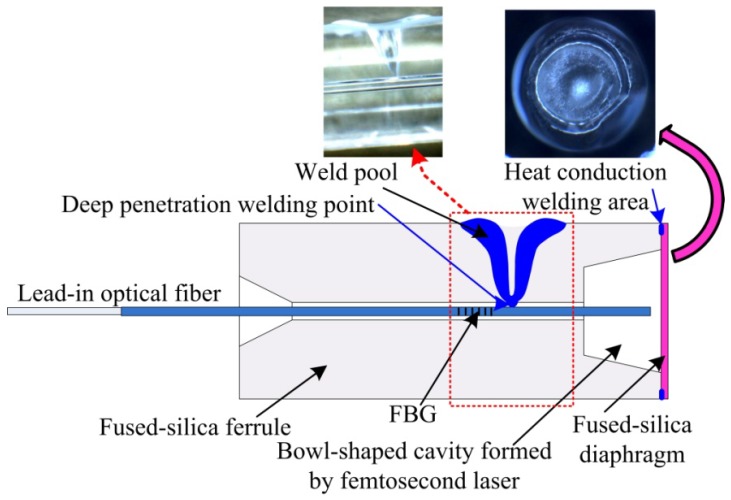
Schematic diagram of the cascaded sensor of the Fabry-Perot interferometer (FPI) and the fiber Bragg grating (FBG); inset on the left: deep penetration point welding between the single mode optical fiber (SMF) and the ferrule; inset on the right: heat conduction welding of diaphragm.

A cascaded sensor based on FPI and FBG is shown in Figure 1. The optical fiber end face and the inside surface of diaphragm constitute the two mirrors for the Fabry-Perot (FP) cavity. Light from the optical source propagates to the end face of SMF and is partly reflected, forming a reference light for the FPI, and then it propagates to the inside surface of the diaphragm and is also partly reflected, forming a sensing light of the FPI. The sensing light reflected back is coupled into the SMF and forms interference spectra with the reference light. The FP cavity length, *L*, is obtained by demodulating the interference spectra via a Vernier demodulation algorithm. For the FPI fiber sensor, the diaphragm will deflect due to the applied pressure. If the diaphragm is clamped in a rigid, round shape, the deflection of the diaphragm center, *Y*, under the applied pressure, *P*, can be expressed as [28]

(1) $$Y=\frac{3\left(1-\mu^{2}\right)P}{16Eh^{3}}{\left(a^{2}-r^{2}\right)}^{2},$$

where *μ*, *E*, *h*, and a are the Poisson’s ratio, the Young’s modulus, the thickness, and the effective radius of diaphragm, respectively, and *r* is the radial distance to the diaphragm center. The *L* value will change with the deflection of the diaphragm when the environmental pressure is applied to the FPI sensor. Therefore, the interference pattern of FPI is modulated by the applied pressure, which will be used for the demodulation of the *L* value. After determining the material of the sensor, the sensitivity of the diaphragm-assisted FPI sensor, *Y*/*P*, depends on *h* and *a*. The desired sensitivity can be obtained by designing suitable *a* and *h* according to practical application and the preparation process.

The effective refractive index and the grating period of the FBG will vary when the temperature changes, so the Bragg wavelength, *λ_B_*, is sensitive to temperature. The *λ_B_*-shift, Δ*λ_B_*, with temperature change, Δ*T*, can be expressed as

(2) $$\Delta \lambda_{B}=2\left(\Lambda \frac{\partial n_{eff}}{\partial T}+n_{eff}\frac{\partial \Lambda }{\partial T}\right)\cdot \Delta T,$$

where Λ, *n_eff_*, and *T* are the period of the FBG, the effective refractive index, and the environmental temperature. Accordingly, temperature variation can be simply acquired with the FBG by means of measuring the Δ*λ_B_* of the FBG. The *λ_B_* can be directly obtained by a peak search algorithm. 

### 2.2. Fabrication of Seepage Pressure Sensor

**Figure 2:**
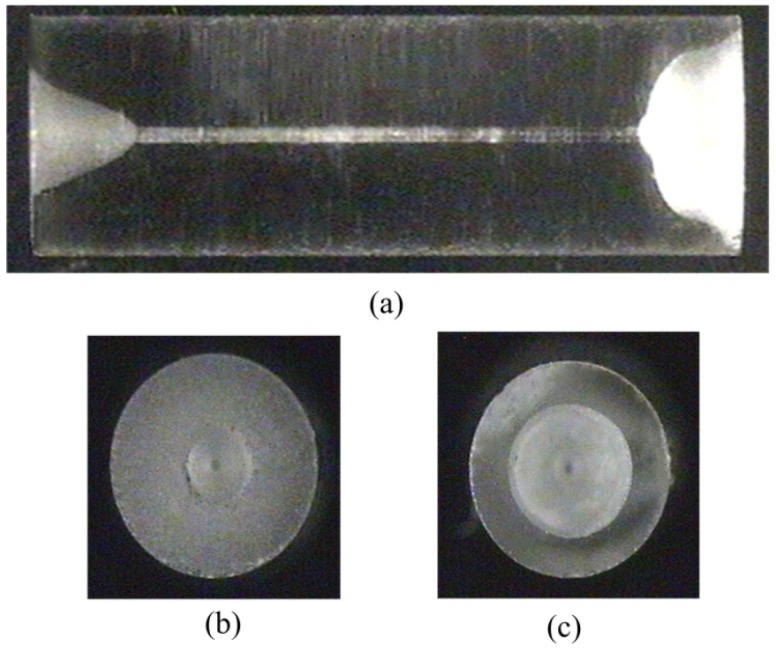
Photo micrograph of the fused-silica ferrule: (**a**) side view, (**b**) and (**c**) top view of end face for the conical cup and bowl-shaped pit in the non-conical end face.

**Figure 3:**
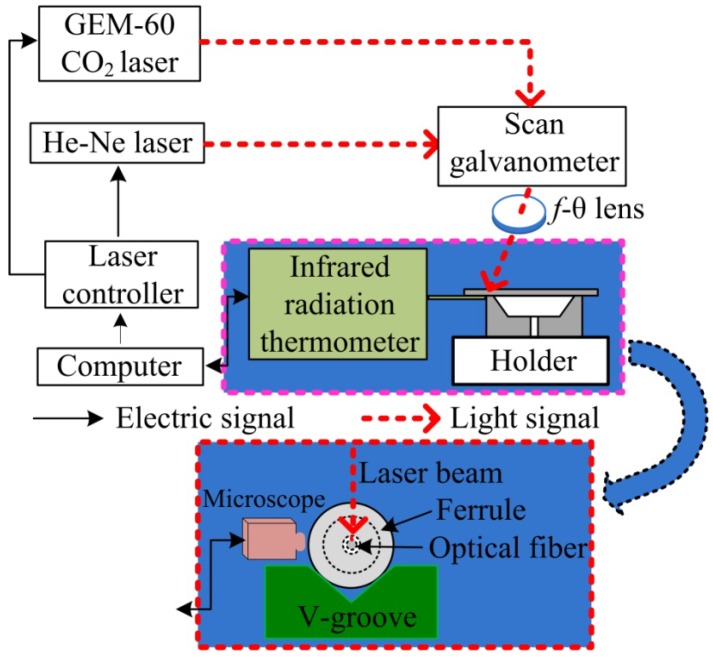
Laser welding system for fabrication of the FPI sensor. The pink dotted frame is for the heat conduction welding of diaphragm, while the red dotted frame is for the deep penetration point welding between the SMF and the inner wall of the ferrule.

The seepage pressure sensor consists of all-fused-silica materials, which are, respectively, SMF, a fused-silica ferrule with a conical cup, and a fused-silica diaphragm. The fused-silica ferrule is shown in Figure 2a–c. Figure 2a shows a side view micrograph, and Figure 2b depicts a top view micrograph of the conical cup end face. We used a femtosecond laser to fabricate a bowl-shaped pit in the non-conical end face, and the top view micrograph is shown in Figure 2c. The sensor was fabricated by CO_2_ laser (GEM-60, Coherent Inc., Santa Clara, CA, USA) welding, and the laser welding system is shown in Figure 3, where the pink dotted frame is for the heat conduction welding of the diaphragm, while the red dotted frame is for the deep penetration welding between the SMF and the inner wall of the ferrule. The diaphragm and the SMF were welded, in turn, to the end face and the inner wall of the ferrule. First, we welded the diaphragm onto the bowl-shaped end-surface of the ferrule by laser heat conduction welding. During welding, the ferrule and the diaphragm remain stationary while the laser beam rotates at 500 mm/s for 1000 circles. Next, we inserted a SMF connected to a sm125 (Optical Sensing Interrogator, Micron Optics Inc. Atlanta, GA, USA) into the through-hole of the ferrule, and then a FP cavity was constructed by the end-face of the SMF and the inside surface of the diaphragm. The FP cavity length was designed to be approximately 115 μm and is monitored in real-time by the sm125. The L value was calculated by our Vernier demodulation algorithm. At last, we fixed the SMF to the inner wall of the ferrule by laser deep-penetration point welding. During welding, the ferrule, the SMF, and the laser beam remain stationary. Laser deep-penetration welding causes a keyhole effect until the SMF is fixed on the inner wall of the ferrule. The microscope in Figure 3 is used to monitor the welding between the ferrule and the SMF. A schematic diagram of sensor is shown in Figure 1, where the inset on the left shows the deep penetration welding point of SMF, and the inset on the right illustrates the heat conduction welding of the diaphragm. The all-fused-silica structure for FPI can avoid the performance decline of the sensor due to materials pressing against each other due to the difference in CTE.

## 3. Experiment and Results

**Figure 4:**
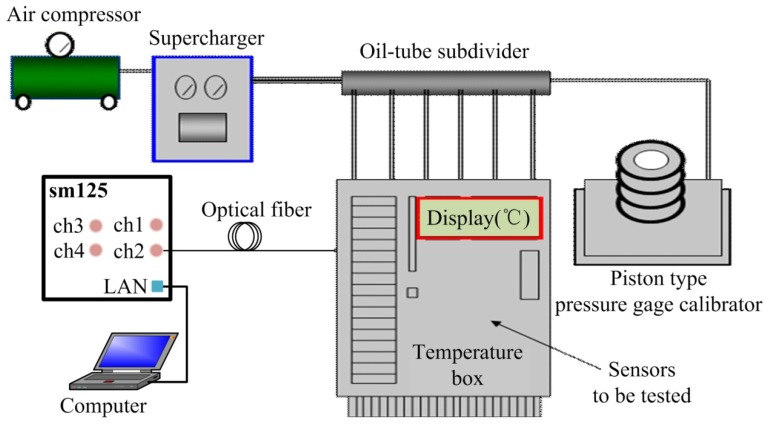
Calibration/test system for the pressure/temperature of the FPI/FBG multiplexing sensor.

**Figure 5:**
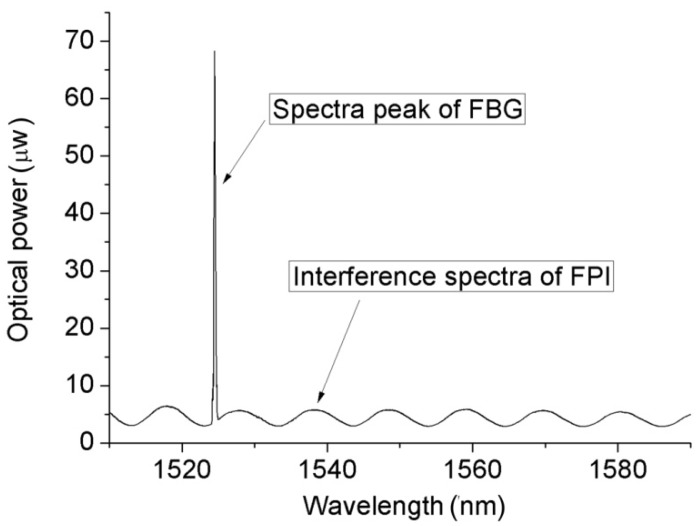
Illustration of the mixed spectra of the FPI/FBG multiplexing sensor.

Figure 4 depicts the experimental system that was used to determine the pressure characteristics of the FPI sensor at different temperatures and to test the temperature characteristics of the FBG. The mixed FBG and FPI sensor was placed into a temperature furnace together with a platinum resistance thermometer. The temperature was displayed on a thermometer 1502A (Fluke Co., Everett, WA, USA) with an accuracy of 0.2 °C. The environmental temperature obtained by the FBG was used to compensate for the pressure measurement errors of the FPI sensor due to temperature variation. The light beam emitted by the sweeping laser in the sm125 was launched into the SMF, and then reached the FBG and the FPI in turn. Also, the sm125 was employed to monitor the returned spectra with an accuracy of 4 pm and a wavelength range of 1510–1590 nm, and this was displayed on the personal computer. The reflected mixed spectra of the FPI and the FBG are shown in Figure 5, where the spectrum peak corresponds to the FBG reflection wavelength and the periodic interference spectrum was produced by the FPI. The FP cavity length was calculated by the Vernier demodulation algorithm.

**Figure 6:**
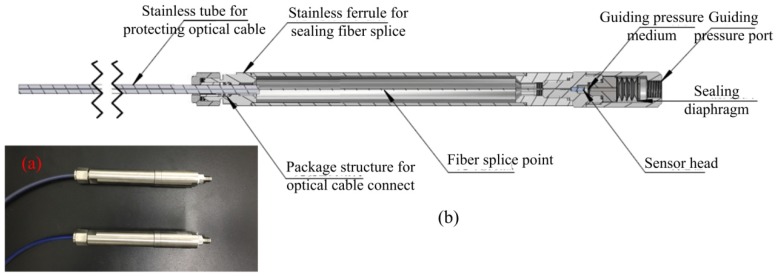
(**a**) The packaged FPI/FBG sensor and (**b**) its packaged structure.

**Figure 7:**
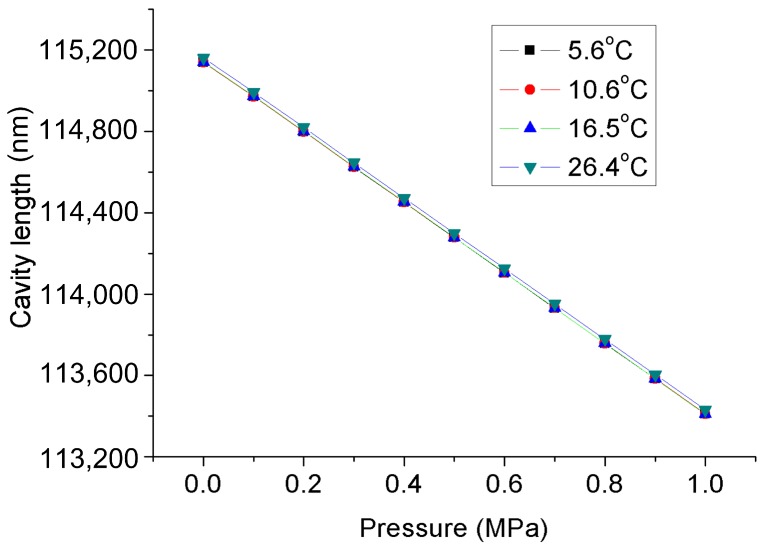
Pressure response characteristics of the FPI/FBG seepage pressure sensor at different temperatures.

**Figure 8:**
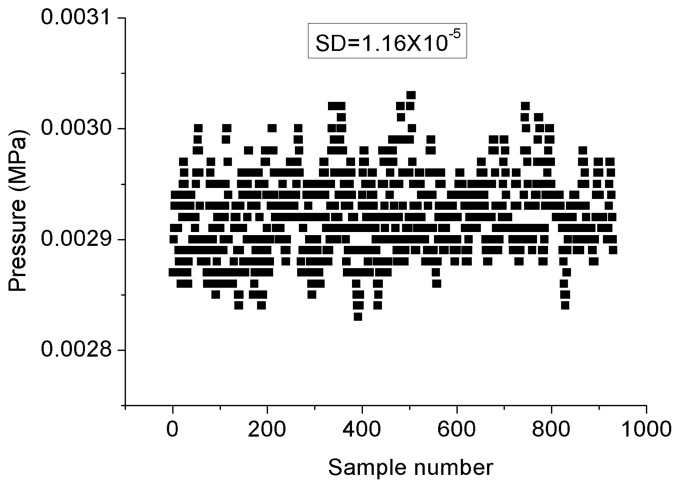
Measurement standard deviation of the FPI sensor.

As described above, the FPI was used to measure the seepage pressure, while the FBG was only designed to measure the temperature of the environment. The pressure acting on the diaphragm was supplied by a hydraulic piston pressure gauge (3100 series, General Electric Co., Boston, MA, USA), and the pressure guide port of pressure conduction connector was connected to the oil tube for the hydraulic gauge, as shown in the test system depicted in Figure 5. The packaged sensor and its package structure are shown in Figure 6. The pressure characteristics of the sensor were demonstrated from 0 to 1 MPa with an interval of 0.1 MPa at different temperatures, and the temperature characteristics of FBG were measured from 26.4 to 5.6 °C. The temperature in the levee and the dam body is affected by the reservoir water, and the temperature is usually near or below the ordinary temperature. The seepage pressure response characteristics of FPI are shown in Figure 7 at 26.4, 16.5, 10.6, and 5.6 °C. The results show that the pressure of the FPI sensor has good linearity at different temperatures, and the sensitivity is about 1.732 nm/kPa. However, the seepage pressure has slightly differences at different temperatures due to the temperature-pressure cross-sensitivity of the sensor. The cross-sensitivity will lead to measurement error for pressure when the environmental temperature varies. The change in FP cavity length is shown in Figure 8 at 15 minutes at 0 MPa, and its standard deviation is 1.16 × 10^−2^ kPa. We used twice the standard deviation as the resolution of the FPI sensor, so the resolution of the sensor is 2.3 Pa.

**Figure 9:**
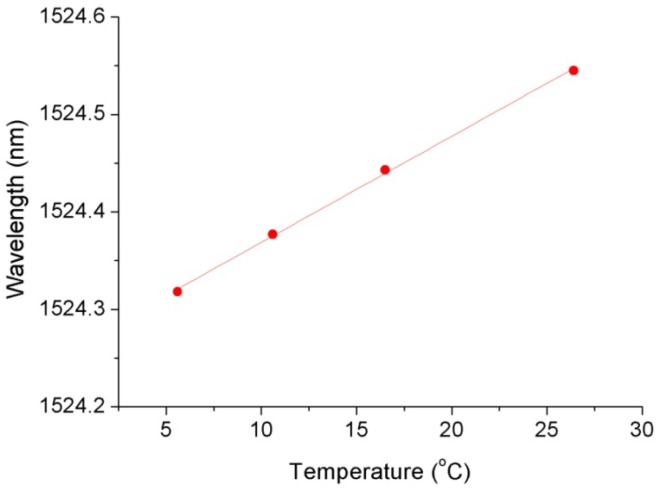
Temperature characteristics of the FBG.

**Figure 10:**
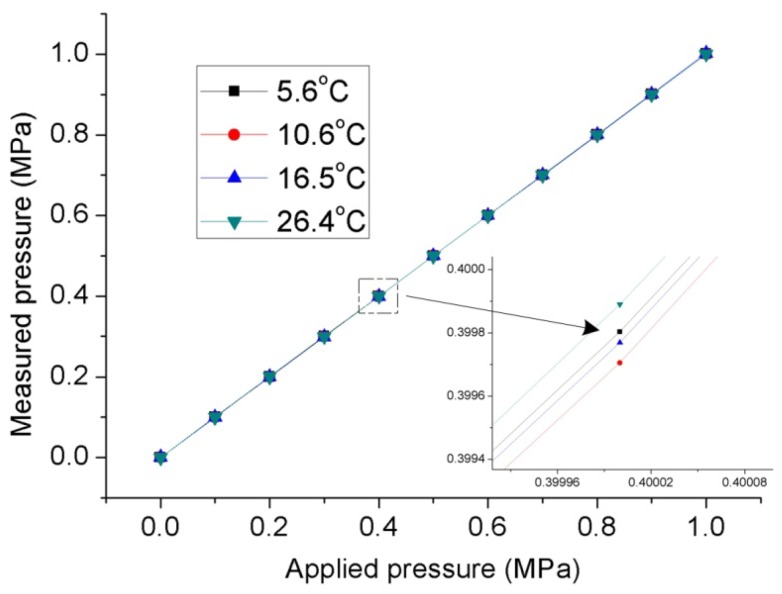
Calibration curves for the FPI/FBG pressure sensor at different temperatures after temperature compensation; inset: enlargement at 0.4 MPa.

In order to reduce the measurement error caused by the difference in pressure response at different temperatures, we used the environmental temperature obtained by the FBG for temperature compensation of the FPI. The temperature calibration curve of the FBG under atmospheric pressure is shown in Figure 9, and the temperature sensitivity is 0.011 nm/°C. After temperature compensation, the calibration curves of the FPI sensor at different temperatures were as shown in Figure 10. The seepage pressure measurement deviation caused by temperature-pressure cross-sensitivity of the FPI sensor was well compensated.

**Figure 11:**
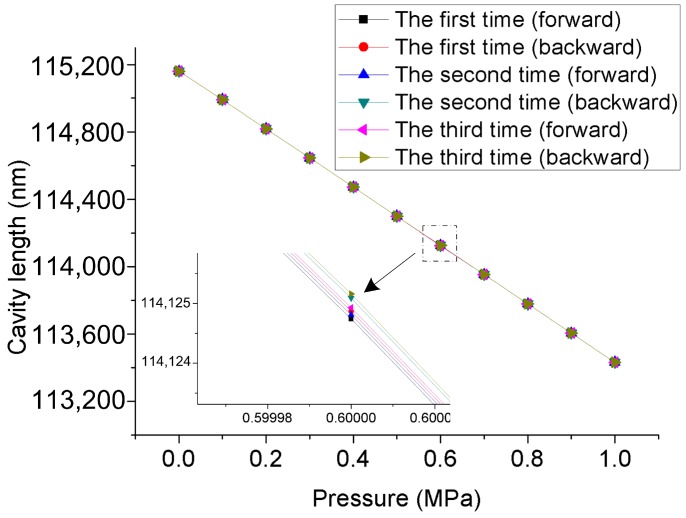
Hysteresis for the FPI/FBG seepage pressure sensor; inset: enlargement at 0.6 MPa.

Hysteresis is an important performance factor for high accuracy monitoring of the seepage pressure of levees and dams. The measurement of pressure at a normal atmospheric temperature was continuously repeated for three cycles of forward and reverse travel, and the results are shown in Figure 11. The results indicate that the hysteresis error of the sensor is very small, and the maximum pressure deviation is 0.21 kPa in the range of 0–1 MPa.

**Figure 12:**
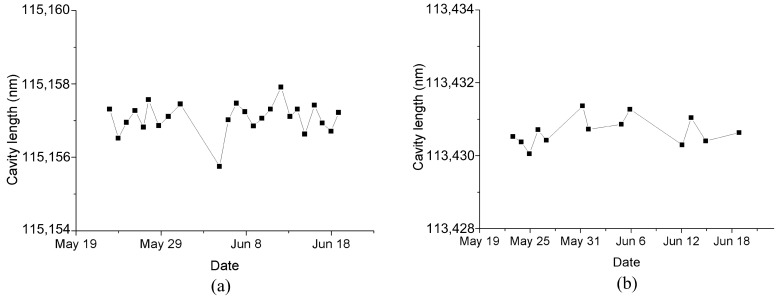
Long-term stability of the FPI/FBG seepage pressure sensor at (**a**) 0 MPa and (**b**) 1 MPa.

**Figure 13:**
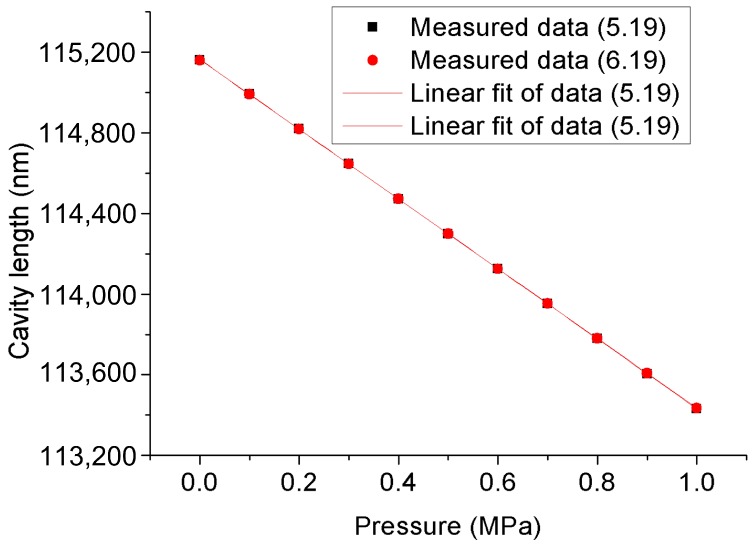
Pressure response characteristics of the FPI seepage sensor on May 19 and June 19.

To verify the long-term stability of the FPI sensor, we recorded the changes in cavity length over time each day, and the daily cavity length values are the result of the average cavity length measured at one hour intervals. Figure 12a,b depict the long-term stability of the FPI sensor for about one month at 0 MPa and 1 MPa, respectively. It can be seen from Figure 12 that the cavity length of the FPI sensor has good stability. The maximum pressure fluctuation is about 0.8 kPa, and there is no change trend in a single direction. Additionally, we interrupted the data acquisition on May 31 and resumed the data acquisition on June 5. Before re-collecting data, we artificially changed the pressure and then quickly recovered it to 0 MPa, so Figure 12a shows that the cavity length has a sudden change between the data for May 31 and June 5. This process of change indicates a sensitive response of the sensor to environmental pressure. The pressure response characteristics of the FPI seepage sensor on May 19 and June 19 are shown in Figure 13. The results illustrate that they are basically in accordance.

## 4. Conclusions

In summary, we presented an optical fiber pressure sensor cascading a diaphragm-assisted FPI and an FBG. The presented sensor consists of homogenous fused-silica materials and was fabricated by CO_2_ laser all-welding, which formed a seepage pressure sensor with a monolithic structure. The monolithic structure makes no difference to the CTE between materials, keeping the diaphragm and the ferrule from pressing each other and improving the performance of the sensor. Moreover, laser deep penetration point welding opens the FP cavity, resulting in there being no problem related to measurement error caused by residual air in the FP cavity squeezing the diaphragm due to temperature rise. The pressure of the monolithically structured FPI sensor has good linearity at different temperatures. We used the cascading FBG temperature sensor to compensate for the difference in seepage pressure responses at different temperatures due to the temperature-pressure cross-sensitivity of sensor. The resolution of the FPI sensor is 2.3 Pa. Additionally, the monolithically structured sensor possesses excellent hysteresis and long-term stability. The results show that both its resolution and stability are better than those of previously reported sensors using monolithic sapphire and stainless steel. The proposed sensor could be widely used to sense pressure in harsh environments, such as damp, corrosive, and high temperature environments due to its all-laser-welding.
